# “Between formulas and freestyle” – a qualitative analysis of peer tutor preparation and its impact on peer relations

**DOI:** 10.1186/s12909-024-06191-7

**Published:** 2024-10-19

**Authors:** Doreen Herinek, Robyn Woodward-Kron, Michael Ewers

**Affiliations:** 1 Institute of Health and Nursing Science, Charité – Universitätsmedizin, Berlin corporate member of Freie Universität Berlin and Humboldt-Universität zu Berlin, Augustenburger Platz 1, 13353 Berlin, Germany; 2 Department of Medical Education | Melbourne Medical School, University of Melbourne, The University of Melbourne, Melbourne, VIC 3010 Australia

**Keywords:** Student peer tutors, Interprofessional education, Peer relations, Peer-assisted learning, Qualitative research

## Abstract

**Background:**

Peer tutorials are widely used in medical and health professions education. Some evidence suggests that peer tutorials can have positive effects for student peer tutors and tutees alike. To promote these positive effects, peer tutors are often prepared for their tasks. However, detailed information about this preparation is missing in the literature. The present study thus examines from the tutors’ perspective how peer tutor preparation is conducted, both in interprofessional and uniprofessional contexts, and how this preparation affects peer relations.

**Methods:**

A qualitative design was used for the study. For data gathering, three online focus group discussions were conducted with student peer tutors from uniprofessional and interprofessional settings who had a background in health professions. Data were analysed inductively via thematic analysis.

**Results:**

A total of 19 individuals participated in three focus group discussions (*n* = 6/*n* = 6/*n* = 7). From the participants’ perspective, preparation measures were heterogenous. Of a wide range of different measures, some were perceived as more helpful, others as less helpful. In analysing the data, three relevant themes came up which were dereived from the peer tutors’ perspective: *roles*, *eye level*, and *(self-)trust*. All three themes were found to be closely related and appeared to have a direct influence on peer relations. This influence on the learning/teaching process was either positive or negative depending on the respondents’ experiences.

**Conclusion:**

From the peer tutors’ perspective, the preparation they received affected their relationships with their peers in a variety of ways, influenced by the context and the peer tutors’ needs. This finding should be taken into account when planning and implementing future preparatory activities. In addition, further pedagogical considerations and discussions about preparatory activities for peer tutors and their potential impact on social and cognitive congruence are needed.

**Supplementary Information:**

The online version contains supplementary material available at 10.1186/s12909-024-06191-7.

## Background

A shift away from teacher-centred teaching formats and towards independent and cooperative learning conducted by trainees and students has been observed in medical and health professions education [1]. This shift can be supported via peer-assisted learning (PAL). PAL is defined as *“[…] a social practice of mutually beneficial personal and professional development among learners who interact as status equals [and that is] characterised by safety*,* comfort*,* motivation through relevance*,* and intellectual risk-taking”* [2, p. 12]. It is characterised by the following important critical factors: (1) Emphasis is placed on learning (rather than teaching), (2) action and reflection take place between learners of equal status, and (3) small groups of learners interact with and support one another without a professional teacher [3–5].

Social and cognitive congruence are key factors often used to legitimise and explain the positive impact of PAL from a learning theory perspective [6–8]. Social congruence contends that those involved in the learning process have similar social roles and can hence exchange information more easily. Moreover, cognitive congruence means that the learners have approximately the same level of knowledge and therefore complement one another (ibid.). The factors that constitute social or cognitive congruence can influence social peer interactions and therefore also peer relations. In this sense, peer relations are understood to be limited in time and thus also to be less close than e.g., friendships [9] while nevertheless remaining interdependent [10].

Empirical evidence of PAL has been documented mainly in medical education. Findings show positive effects for different learning domains (e.g., knowledge acquisition or even practical skill acquisition), in different settings (e.g., clinical settings or universities), and for different groups of participants (e.g., peer tutors and tutees) [11–13]. Comparisons of PAL with instructor-led teaching suggest that PAL can be a resource-saving alternative. In certain circumstances, PAL can also result in equal learning outcomes [14] or even in better learning outcomes than in instructor-led teaching [15].

Peer tutorials are considered a subtype of PAL [16]. They build on the co-operative approach of PAL but offer more structure to support positive learning outcomes. However, there are gradual differences compared to the features of PAL described above. Peer tutors are often one or two years ahead and take a more active role in helping, guiding or supporting their tutees [3]. This aligns with Banduras social-cogintive theory which states that learners interact with more experienced ones and that this promotes self-efficacy, one of the key factors for successful learning. Social interaction is therefore considered a prerequisite for learning and regards learning as a dynamic process [17, 18].

For peer tutorials to be successful, the broad consensus in the literature suggests that it is necessary to train peer tutors in order to improve the quality of their tutorial provision [19–22]. Training is provided to prepare peer tutors for both their current and future role as lifelong teachers and learners in their respective professions [23].

The effects of peer tutor training documented thus far are mostly related to uniprofessional contexts, with the training leading to demonstrable increases in competencies, such as increased didactic and methodological skills [24]. Moreover, peer tutors have been found to gain confidence in speaking in front of and moderating groups [25]. These tutors view themselves as role models and report an increase in judgement and evaluation skills [26]. The need for tutor preparation is supported by research showing that without prior preparation, peer tutors may experience feelings of uncertainty and overwhelmedness when delivering peer tutorials [27, 28]. In sum, highly heterogeneous peer tutor training practices have been reported in uniprofessional contexts. This situation has been documented both at the international level [29] and at the national level for Germany [30]. For the interprofessional context, little evidence is available on the preparation of peer tutors. Herinek et al. (2022a) [30] confirmed that interprofessional tutorials and corresponding preparation remain rare in Germany and that the preparatory measures differ in their aims, scope, and manner of implementation. Furthermore, Homberg et al. (2022) [19] found an increase in tutors’ didactic and methodological teaching skills. Next to this they proved tutors’ positive attitudes towards interprofessional teaching and learning in general and towards the interprofessional learning experience during the training programme in particular.

No systematic investigation into how the preparation of peer tutors is performed or how this preparation affects the tutors themselves has yet been conducted. Moreover, it remains unclear how peer tutor preparation may influence social interactions and peer relations as the main impact factors of this learning format. In particular, the perspective of the peer tutors and their perception of both the tutor preparation and its effects have yet to be captured. Against this background, the present study investigates peer tutors in medical and health professions education by asking the following questions:

1. From the perspectives of the peer tutors, how were they prepared for their tasks?
2. Are there differences in the experiences of how peer tutors are prepared in uniprofessional and interprofessional contexts?
3. What impact did the peer tutors’ preparation have on themselves and on their relationships with the other learners from their point of view?

The study aims to draw conclusions for the future design of tutor preparation from the experiences of the peer tutors with their tutor preparation and its perceived impact on the tutors’ relationships with their peers. Furthermore, it should be possible to make recommendations for future educational research initiatives in this field.

## Methods

The present study is part of a larger project entitled Prep4TUT (Preparation For a Tutoring Activity for (Interprofessional) Peer-Assisted Learning) [31]. An ethics approval for the entire study was provided by the institution with which the first author is affiliated (EA1/270/20). All participants were informed about the handling of their data (anonymity, voluntary participation, data protection, etc.) and were asked to affirm their consent in writing.

### Research design

The study followed an explorative–qualitative design due to the limited empirical evidence and the incomplete or inconsistent state of research and knowledge on peer tutor preparation, especially in an interprofessional context. Focus group discussions (FGD) were used as the method for data gathering [32]. FGD made it possible to explore, synthesise, and elaborate divergent viewpoints among the diverse experiences and perceptions of the interviewees through their interaction as well as to better capture complex phenomena [32].

### Participants, recruitment, and data collection & processing

Peer tutors from Germany were sought who were active in the field of uniprofessional or interprofessional peer tutorials at universities and universities of applied sciences. In principle, health professions had to be qualified, and tutorials had to be offered at the tutor’s institution. For this purpose, a short survey was carried out via email in advance. First, a gatekeeper sampling strategy was applied. Deans’ offices or study and teaching departments were contacted and asked to forward the request for participation to their peer tutors. Second, a snowball sampling strategy was used to disseminate the call for participation via email to professional associations and networks in the field of interprofessional education with the request to forward the email to peer tutors. Additionally, the participating peer tutors were asked to relay the call to other peer tutors they knew. Overall, a deductive sampling strategy was chosen in order to capture the most heterogeneous possible perspectives and experiences. The comprehensive inclusion criteria are summarised in Appendix A.

FGD were conducted from August to October 2022. For data collection, a interview guide was created in line with Krueger and Casey (2009) [33], who suggest using opening questions, introductory questions, transition questions, key questions, and closing questions. In terms of content, the topics were selected based on the literature, and the interview guide was then structured according to Helfferichs’ (2011) [34] SPSS principle: (1) collect (German: sammeln), (2) check (German: prüfen), (3) sort (German: sortieren), and (4) subsume (German: subsumieren). The interview guide can be found in Supplement A.

The FGD were conducted online in German via the platform Microsoft Teams (version 16.2.8) by DH – a female junior researcher with an M.Sc. in Health Professions Education who has a medium level of experience in interviewing – as part of her dissertation project. The FGD were conducted online for pragmatic research reasons in order to involve individuals from different institutions and in different locations within the discussion. DH introduced herself with information on her personal background as well as on her research interest and the objectives of the study. In each case, two additional individuals – both also junior researchers (one with a focus on special incidents – e.g., non-verbal peculiarities – and one who helped in documenting the course of the discussion) – took notes in the digital room. After a short introduction and description of their tasks, the researchers switched off their cameras in order to minimise distractions. Following the discussions, the participants were asked to complete a short questionnaire about their socio-demographic data and previous tutoring experience. In addition, the interviewer filled out a reflexive protocol that recorded, for example, the interview atmosphere, incidents, and the central content of the discussion.

Three online FGD were conducted for data collection. They lasted an average of 1:39 h. A total of 19 individuals took part in the FGD: 6 in the first, another 6 in the second, and another 7 in the third (no dropouts; all who volunteered took part). Table 1 lists the characteristics of the sample, including each individual’s health profession.

**Table 1:** Participant characteristics

	Age	Gender	Discipline		Semester	Duration of tutoring	Experience with interprofessional tutorials
**FGD1** (***n*** = **6**)	23–33 years (Ø 27 years)	female: 3 male: 3	Medicine	*n* = 5	2–12 (Ø 8)	1–3 years (Ø 1.8 years)	Yes: *n* = 5 No: *n* = 1
			Physiotherapy	*n* = 1			
**FGD2** (***n*** = **6**)	22–43 years (Ø 30 years)	female: 3 male: 3	Medicine	*n* = 3	2–9 (Ø 5)	1 month – 5 years (Ø 1.5 years)	Yes: *n* = 6 No: *n* = 0
			Nursing	*n* = 1			
			Occupational therapy	*n* = 1			
			Speech and language therapy	*n* = 1			
**FGD3** (***n*** = **7**)	21–35 years (Ø 28 years)	female: 6 male: 1	Physiotherapy	*n* = 5	2–18 (Ø 6)	6 months – 2.5 years (Ø 1.4 years)	Yes: *n* = 3 No: *n* = 4
			Nursing	*n* = 2			
**Total** (***n*** = **19**)	21–43 years (Ø 28 years)	female: 12 male: 7	Medicine	*n* = 8	2–18 (Ø 6)	1 month – 5 years (Ø 1.5 years)	Yes: *n* = 14 No: *n* = 5
			Physiotherapy	*n* = 6			
			Nursing	*n* = 3			
			Occupational therapy	*n* = 1			
			Speech and language therapy	*n* = 1			

All online FGD were audio-recorded. Data were transcribed verbatim using simple transcription rules in line with Dresing/Pehl (2015) [35] and were comprehensively pseudonymised [36].

### Data analysis

Data were analysed inductively using reflexive thematic analysis in line with Braun/Clarke (2022) [37] with the help of MAXQDA 22 software (VERBI Software GmbH, Berlin, Germany). Reflexive thematic analysis (TA) is characterised by the identification, analysis, and documentation of patterns of meaning in a dataset [37, 38]. In addition, both latent and manifest content can be considered in the analysis and – compared with pure content analysis – lead to an in-depth description of a discovered phenomenon [38]. Overall, a relativist–constructionist theoretical framework was used to conduct reflexive TA, particularly when it came to eliciting the deeper meanings behind peer relations and the interplay between external (e.g., institutional) circumstances and the influence of these circumstances on peer tutors’ individual behaviour [37]. A statement on reflexivity and a detailed overview of the phase-by-phase analysis in line with Braun/Clarke (2022) [37] including proportionate task processing can be found in Appendix B.

## Results

The results of the analysis are presented in two parts. The first part focuses on a description of the participants’ expericences of how they either were prepared or how they prepared themselves for their tutor role in uniprofessional and interprofessional contexts. It also highlights what they perceived as helpful, unhelpful, or missing. These descriptions provide contextual information to understand and situate the results of the subsequent in-depth thematic analysis.

### Experiences with the preparation as a peer tutor

Some peer tutors – mostly those from uniprofessional settings – mentioned that they had been prepared for their work via highly structured preparation programmes. These programmes often included strict procedures: attending a compulsory course, then observing other peer tutors conducting a tutorial, conducting an own practice tutorial, and finally, having a teacher-observed practice tutorial approved by lecturers or more experienced peer tutors. In particular, the opportunity both to have scripts available for preparation and to receive feedback from experienced individuals on their own performance was described positively by most of the participants who had undergone this sort of preparation.

The concept of learning from experienced peer tutors appears to have been valuable in terms of preparing new tutors. This point was emphasised by peer tutors who worked in interprofessional contexts and often received less preparation guidance from their institution. Despite the greater degree of self-determination, structured preparation was found to be helpful, as were informal opportunities for discussion with individuals from other disciplines. First, all key materials were available online for independent reading. Moreover, opportunities for observation as well as independent and supervised tutorials also came into play. Participants generally appreciated when contact people were identified and available for questions during the preparation phase or when professional idea exchange with others was possible. All participants reported preparing themselves in some way for their role by either receiving formal preparation or undertaking the preparation on their own. Such individual preparation included independently acquiring knowledge through reading, deepening what had already been learnt, acquiring new methodological skills, or even (further) developing (new) tutorials.

In summary, tutors in uniprofessional contexts usually received highly structured preparation from the institution, which was geared towards the content to be taught and its evaluation, as well as towards the didactic and methodological design of tutorials. In contrast, tutors in interprofessional contexts often took their preparation into their own hands; this was less content-oriented and more focused on facilitating groups.

### Description of themes

**Fig. 1:**
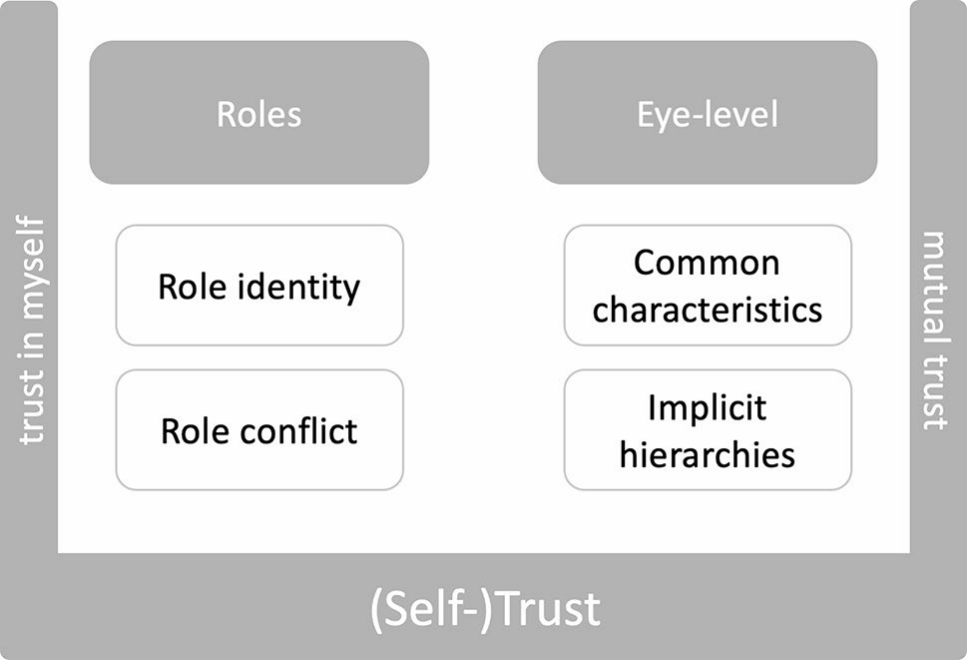
Thematic overview

Our reflexive thematic analysis identified three themes in the data as relevant to the research questions: (1) *roles*, (2) *eye level*, and (3) *(self-)trust*. These themes are presented below along with their respective sub-themes (see Fig. 1), and their dimensions are set out.

### Theme 1: Roles

Peer tutors who participated in the study reported being aware of taking on a certain role as a peer tutor that entailed specific demands. They stated that their preparation had given them the chance to *“grow into the tutor role”*^1^*(Occupational Therapy student*,* FGD2; Medicine student*,* FGD2)*, and they considered this growth necessary. The interviewed peer tutors linked their actions to the demand for a certain form of behaviour, appearance, and interaction, and they perceived themselves in different roles depending on the intention of the tutorial.

### Role identity (sub-theme)

The FGD participants were unsure which role they wanted to take on for themselves, and the question of “Who am I?” was raised. They expressed uncertainty about their role identities, e.g.; they found themselves in a “basically intermediary” role (two Public Health students, FGD3) which was explained as follows: the tutors perceived themselves as being somewhere between the group of learners for whom they felt responsible and the lecturers or institutions for which they worked as peer tutors (i.e., for which they were employed). Hence, depending on the context, the role was fulfilled differently by the participants: The more the tutorial aimed at imparting knowledge and exam preparation, the more the role of teacher or “expert” was assumed (which was mainly the case in uniprofessional contexts). However, when the tutorial had other main aims, the participating peer tutors felt more as facilitators and organisers, and sometimes also as arbiters of disputes or simply as learners in the same way as their tutees (mainly in interprofessional contexts). The role of the peer tutor was thus interpreted differently in these two contexts. The peer tutors from interprofessional learning groups in this study viewed it as their role to create spaces for idea exchange and intervening – if at all – as moderators:

*“[…] That means that I had no pressure to be an expert on a particular topic*,* and it was more about facilitating and basically making sure that a conversation was taking place and that everyone was engaged with in some way. And that definitely made it very easy for me to get started because I didn’t feel like I was under a lot of pressure. Instead*,* I felt like I was on eye level with the participants.” (Physiotherapy student*,* FGD3)*.

This quotation represents the different focus that interprofessional tutorials were found to have. For these tutorials, focus was mostly not placed on teaching facts or skills, which the participants found to be a great relief. Consequently, the peer tutors described different approaches to the tutorials and felt more comfortable dealing with interpersonal issues. Peer tutors for uniprofessional learning groups viewed themselves more as guides, input providers, and role models. They described their role as one of teaching content, preparing for exams, and achieving the goals they had been given, which is why great importance was attached to carefully preparing these tutorials.

### Role conflicts (sub-theme)

Role conflicts experienced by students arose mainly from the fact that the peer tutors not only perceived themselves in various roles; instead, they were also assigned by the tutees or by the institution, e.g., as part of the preparation.

*“Generally*,* the teachers do their thing amongst themselves*,* and then*,* the tutors are asked*,* ‘How did it go for you?’ And then*,* you get into this stupid dilemma of thinking*,* ‘OK*,* you’re on the same level [as the tutees]*,* and at the same time*,* you’re supposed to give an assessment’*,* which you don’t really want to do because it totally invalidates that eye-to-eye thing. And that is basically always a really uncomfortable position [to be in]*,* I find.” (Public Health student*,* FGD2)*.

These role conflicts – according to the opinion of the interviewed tutors – always involved some form of assessment of tutees, be it as verbal feedback to the lecturers (as in the quotation above) or as an actual assessment of examination performance:

*“And at that time*,* for example*,* I actually had to make assessments. I had to look at exams*,* look at tests*,* even talk to the main lecturer. But that’s not nearly as much [work] as it used to be. So I got closer to the lecturer. So I grew a bit distant from my tutees*,* if I can put it that way.” (Nursing student*,* FGD2)*.

Some of the participants seemed to feel pressured into taking on such tasks, but in many cases, they did so because they were dependent on their jobs and supervisors.

Due to the intensive compulsory preparations, the student peer tutors were expected to be competent enough, for example, to answer questions in detail and to deal with group dynamics appropriately, as some participants reported. The role that peer tutors assumed due to external constraints had an influence on how *close* they felt to their tutees:

*“The very fact that I was filling this role separated me from the group even though I was still part of the group. But because of the role*,* I was different. (Nursing student*,* FGD3)*

However, the preparation itself as well as the external expectations did not necessarily influence the tutors’ view as their tutees’ peers. Indeed, a few peer tutors still defined themselves as *“peers with a different function and in a different role” (Nursing student*,* FGD3)*. The effects of such a perspective – also against the background of the peer tutors’ preparation – on the formation of relations between tutors and tutees is explained below.

### Theme 2: Eye level

The peer tutors who participated in the FGD repeatedly used the term “eye level”, which is best understood through the concept of “closeness and distance” between peer tutors and tutees. The tutors felt that these two characteristics were not purely dichotomous and overlapped significantly.

### Common characteristics (sub-theme)

The extent to which peer tutors perceived themselves to be on the same level as their tutees depended significantly on the characteristics they have in common. For example, tutee status and similar experiences were mentioned as characteristics of proximity. In contrast, privileges – such as access to rooms and materials to which the tutees did not have access – and knowledge advantages were mentioned as characteristics of distance. As one tutor stated, *“I think the advantage of [having greater] knowledge is significant in a classical tutorial” (Physiotherapy student*,* FGD3)*. Here, “classical” was used to refer to a uniprofessional tutorial that was primarily concerned with the transfer of knowledge or skills and that prepared tutees for examinations. The participants recognised that once they had been prepared with this focus, there could no longer be an “eye to eye” level of knowledge:

*“And in this respect*,* I actually decided that my qualification means I can really no longer be on eye level [with my tutees] or have the same status that I would perhaps like to. I can try*,* but I simply have different knowledge… maybe even an advantage*,* in my situation.” (Nursing student*,* FGD2)*.

The situation became difficult when dependencies were created between tutors and tutees due to the tutors’ requirement to provide formal assessments. Some participants commented that their lack of involvement in an assessment had allowed for greater closeness between themselves and their tutees. The point was to create an anxiety-free space for sharing that encouraged asking questions (Medicine student, FGD2) and communicating openly (Medicine student, FGD1). If peer tutors were preparing for exams or otherwise assessing their tutees, this impacted the openness of the tutees, as some of the participants stated. They mentioned that they may have been inhibited from speaking at all or felt shameful about not knowing something.

The advantage of having greater knowledge and the requirement to make judgements played less of a role for the peer tutors involved in this study, especially in interprofessional tutorials. Instead, the participants concluded that despite their different professional backgrounds, there was more of a sense of being on equal footing. This was explained by the fact that all participants were still unaware of the other professions and everyone learned from one another. The mutual learning effect was particularly emphasised in this context:

*“But because everything is so interprofessional*,* I always have to be a learner because people from other professions join in who have knowledge that I don’t have and will never have. And because of that*,* I’m in a different position from the very beginning.” (Medicine student*,* FGD1)*.

This statement highlights the interprofessional context as something that could have a positive influence on remaining at “eye level” with tutees because each person involved could contribute to the success of the tutoring. However, the peer tutors’ perspectives underlined the notion that the experiences and informal knowledge about the specifics of the study programme could not be easily shared, thereby potentially leading to a greater sense of distance between tutor and tutee.

### Implicit hierarchies (sub-theme)

The FGD participants often mentioned the desire to be perceived as competent and to represent their own profession well in front of members of other professions. This latter aspect was particularly evident in interprofessional contexts. When the peer tutors were not from the medical profession but had representatives from this profession in their tutorial, feelings of insecurity combined with a desire to avoid embarrassment quickly arose:

*“ […] I teach medical students or give tutorials*,* but I’m not actually a medical student myself*,* so of course I want to represent my profession well and show that I’m well versed in the subject and that I can also teach it well or that I learnt it well at school […].” (Physiotherapy student*,* FGD1)*.

The fear of being seen as incompetent was a recurring theme in the FGD. Some participants felt that their often-personal and content-oriented preparation contributed to the appearance professional competence, especially when medical students were involved and the peer tutors did not belong to this profession themselves. This can be viewed as an attempt by some of the FGD participants to meet members of the medical profession at eye level.

### Theme 3: (Self-)Trust

The two themes presented above have some connections with the theme of trust, which is inherent in almost the entire dataset. This theme involves building trust in oneself (self-confidence) but also in others (interpersonal and system trust), maintaining it, and not letting it down. Various aspects of the latter form of trust have already been addressed: On the one hand, the discussions revealed that various expectations are attached to the role of tutor, and on the other hand, tutors were found to attempt to actively build trust with their tutees, for example, by emphasising commonalities or trying to reduce hierarchies. However, as the impact on self-trust predominated the discussions, it will be explained in more detail here.

Depending on the intensity, degree of structuring, level, and intention of the preparation, peer tutors reported different effects on their individual self-confidence. In most cases, the focus was on the fact that the preparation was helpful in building sufficient abilities for carrying out the tutoring successfully. For example, the tutorials were found to offer orientation, support, and the possibility both to reference what had already been learnt and to re-read things. Moreover, tutorials were also found to help reduce anxiety and the feeling of *“having to stand there empty-handed” (Physiotherapy student*,* FGD3)* or of *“having been left alone” (Physiotherapy student*,* FGD3)*. Due to these effects, the majority of the peer tutors involved in the present study claimed that tutorial preparation was indispensable. Hence, depending on the topic and the aim of the tutorial, they preferred concrete preparation that dealt with practical, complex skills and that could only be learnt through good tutoring in a safe learning environment. The tutors felt that the nature of the preparation is thus intended to help build genuinely practical skills and should not be limited to the written material available. A few voices also felt that their flexibility in designing the tutorial had been curtailed by overly strict guidelines, and they even reported having felt insecure.

Nevertheless, the peer tutors in the study made a clear call for preparing tutors who are new and have not yet had any experience. They stated that at least an *“introduction”* to the didactic–methodological basics is needed in order to provide peer tutors with self-confidence in carrying out their work:

*“I was in my third semester*,* and we didn’t really get anything [to help us]. I would have liked to have a… let’s call it a guide or something like that. Or just*,* yeah*,* ‘guidelines’ always sound stupid… but a bit of an orientation on how to organise everything*,* even if it was only an introduction with*,* ‘Here you go*,* you can do individual work*,* partner work*,* group work. These are the pros and cons. These are the absolute basics that you might need.’ […]” (Physiotherapy student*,* FGD3)*.

This tutor’s cautious choice of words and explanations suggest that even basic – perhaps merely general – didactic–methodological preparation can be sufficient for strengthening tutors’ self-confidence. This notion was similarly agreed upon by other peer tutors.

Experienced peer tutors were less likely to wish for formal preparation. Through “trial by error” and constant reflection, they had practised and gained greater and greater self-confidence and thus required less overall preparation:

*“[…] I could imagine always being prepared for every topic. I think that’s important*,* especially when [the tutoring is] just a matter of conveying facts*,* which sometimes happens when there are [many] rows of students. But […] I could imagine that if I were in a completely different group or in a completely different setting*,* I might prepare less intensively […] because the experience of the tutorials that I’m doing now has helped me think*,* ‘OK*,* you’ll manage somehow’*,* and maybe it’s better sometimes to react to the group and see what the energy or the mood or the atmosphere in the group is like.” (Medical student*,* FGD2)*.

The participants often made a distinction between two elements: on the one hand, how to handle the group and flexibly react to spontaneous developments, and on the other hand, how to design the content or foster practical (clinical) competences. Preparation was found to play a different role and to have a different influence on self-confidence depending on the tutorial’s aims. Based on the FGD, the desire for intensive, structured preparation was found to be greater in tutorials with a content-related/practical focus – possibly even with exam-relevant topics. In contrast this is not necessary in tutorials which focus more on idea exchange among participants, as is the case in interprofessional learning.

## Discussion

The present study investigated preparation practices for student peer tutors in uniprofessional and interprofessional contexts as well as the impact of these practices on peer-assisted learning from the peer tutors’ perspective. Our results indicate that preparation influences not only the learning outcomes of tutors and tutees, but also the relationships between the two groups with some variations between uniprofessional and interprofessional contexts. This finding is significant in light of the impact factors of PAL: This special pedagogical format ideally takes place as an interaction between individuals of the same status in a safe learning environment in which the motivation to participate and learn is maintained [3–5, 12]. In order to achieve these effects, it is important – according to the results of this study – that eye-level is maintained between the tutors and tutees, and that trust is established between the group members. These social dimensions of this teaching–learning format can be influenced and adapted through preparatory measures. This applies in particular to peer tutorials as a sub-form of PAL as the peer tutors often undergo more or less intensive preparation for their role.

When it comes to the question of how the tutors were prepared for their tasks as tutors from their point of view, the FGD participants revealed that heterogeneous practices are used and that there is no consensus around what this preparation should involve. This finding is consistent with the results of one international review study [29] and also with those of two German studies [30, 39] that revealed a plethora of activities when it comes to peer tutor preparation. This result indicates a possible lack of agreement among medical and health professions educators on the content, duration, and structure of such preparatory activities. However, there may be sound reasons for divergent preparation practices because institutions are likely to tailor interventions to their specific settings in order to achieve their individual peer tutoring goals [40, 41].

Next to this and considering the experiences of the participants of this study, tutor preparation should not be overly restrictive, but it should at least provide some guidance. To date, little evidence exists in the literature on the needs of peer tutors in terms of preparation. Instead, some studies have pointed to this very gap [11, 42]. However, based on their analysis of five tutor training programs in Germany, Alvarez/Schultz (2019) [43] concluded that institutions are now tending to move away from rigid preparation and towards needs-oriented measures, though the authors did not indicate how this shift in tutor preparation is being accomplished in concrete terms. Our study underscores the notion that it might be useful to consider tutors’ needs, possibly in a participatory process that may also involve dealing with conflicting expectations. What one peer tutor described as a form of preparation that lies somewhere *“between formulas and freestyle” (nursing student*,* FGD3)* points to a discrepancy that is evident from the different tutor perspectives in our study. While some participants of the FGD were found to be in favour of structured preparation (“formulas”), other opinions – especially those of peer tutors in interprofessional settings – tended to suggest that no preparation at all was necessary in order to remain flexible in their pedagogical decisions (“freestyle”). More research activities on this topic could provide information on how to deal with these diverging expectations.

This study had a special interest in the the impact of the preparation on the peer tutors as well as on peer relations. The three themes identified in the data analysis (*roles*, *eye level*, and *(self-)trust*) are closely interwoven and highlight the social dimensions of PAL.

With regard to the theme roles, our findings suggest that on the one hand peer tutors perceive themselves in different roles and that they on the other hand see themselves confronted with differing role expectations from the institutions’ and tutees’ side. Something similar can be found in the literature, for example, when a tutor in a biology class is reported to act equally as an “authority figure, motivator, and friend” [44, pp. 325–326]. These three exemplary roles alone, to each of which different behaviours are attached, indicate that role conflicts can arise. One of the key findings of our study in terms of role conflicts should be emphasised here. According to our participants, difficulties arose when the preparation was focused on a predominantly input-oriented way and was overly oriented towards the role of knowledge mediators right up to the role of an examiner. This latter aspect was often mentioned in our study by tutors working within uniprofessional contexts: while they are given evaluation sovereignty, there is little room for contradiction on the part of the tutee. This is in violation of above-mentioned impact factors, especially the requirement for social congruence, according to which there should be – inter alia – room for differences of opinion between peers [6–8]. Our results indicate that the tutors desire to do justice to their employer (the institutions/supervisors) and fulfil the assessment task, but at the same time, they find difficulty in having to assess their peers. Solomon/Crowe (2001) [45] concluded that it is difficult for peer tutors to separate the role of assessor from that of learning facilitator. They therefore recommended that this topic be taken up when preparing peer tutors and that it be kept constantly in mind [45].

Despite the challenges mentioned, the tutors in our study emphasised that preparation is important to them to be able to fulfil their tasks competently and to arrive at their role as a tutor. In that way it is important to remember that peer tutoring means that tutors take on a new social role [46]. Settling into this role and gaining clarity about the associated expectations are important aims for student tutors, and preparing these tutors could be an appropriate, supportive means of achieving these aims [47]. As Bandura (1999) stated, *“If people are to work together successfully*,* the members of a group have to perform their roles with a high sense of efficacy”* [18, p. 227). Although researched in other contexts, such as in sports teams [48], work teams [49], or nursing practices [50, 51], different studies support the idea that the clearer a role is, the more effectively it can be performed. In other words, if there is role ambiguity about one’s own role, the effectiveness with which the role can be fulfilled may be minimised [48, 50, 51].

In addition to possible role conflicts, the theme eye level became evident in our analysis as an important issue for relationship-building in the peer tutorials. The literature characterises peer relations among students in PAL settings, including peer tutorials, as lacking in hierarchical structures in terms of status and mutual respect [52]. However, our study introduces a more nuanced perspective. Our participants emphasised that it is important to share common characteristics, such as knowledge or the same field of study, in order to build peer relationships. However, they also recognised that preparation can have potential negative influence, for example, by too much increasing the knowledge advantage. This does not mean that the tutors and tutees have to be identical, but rather close in these characteristics. Also this literature supports that when peer tutors and tutees share roughly similar characteristics, an environment of mutual understanding and collaboration can be fostered because both parties can relate to each other’s experiences and perspectives [6–8]. Again, this links back to Banduras social-cognitive theory, emphazising social interaction as a perquisite of learning [17, 18].

Contrary to the above, specifically the peer tutors in interprofessional tutorials mentioned that peer relations were not completely free of hierarchy. Our results reveal that peer tutors from non-medical courses feel particularly insecure when they have to lead tutorials with medical students. The fact that stereotypes, hierarchies, and power imbalances often present themselves in such a way that physicians are perceived as the dominant profession is already well known from research on the topic [53, 54]. This is true not only for PAL [55], but also for traditional teaching formats [56] and especially in interprofessional practice [57, 58]. However, interprofessional education has been proposed as a potential remedy to such imbalances [59]. It might be important to sensitise tutors in interprofessional settings to such power imbalances and hierarchies in the health workforce and to prepare them to deal with the associated risks for PAL.

On the subject the theme (self-)trust, our data confirm that preparation can positively influence confidence in one’s own ability to conduct a tutorial effectively, which is also fundamental to building a trusting relationship with tutees. This finding is in line with results from other studies that have found that trust is necessary for productive peer relations [44] and is a prerequisite for successful peer tutoring [60]. The discourse on trust has made abundantly clear that trust does not exist without preconditions, that it must be developed, and that it is always associated with vulnerability. One person gives trust, and another tries not to betray it [61, 62]. Hence, trust is part of an active process – i.e., it is changeable depending on external or internal factors [61, 62]. Our study indicates that obstacles exist when it comes to building mutual trust. For example, an internal factor can be the fear of not feeling competent enough and of embarrassing oneself, which points to the tutor’s self-confidence. In this regard, studies have proven that the manner of preparation can have a strong impact on tutors’ self-confidence [63, 64]. This has been found both in our study and in other studies, for example, when tutors indicate that they are more confident in trying out their skills and that they feel competent in terms of the tutorial content [63, 64]. Similarly, Alvarez/Schultz (2019) [43] discovered that preparation primarily affects tutee responsiveness. Tutors are then less afraid to address things, to give tips [43], or to speak in front of the group [25]. Regarding trust-building among peer tutors and tutees, other studies have described the idea that trust is important in peer relations and that it must be developed and built both sides [10, 60–62]. For this process to succeed, there must be a safe space for reflection on the high (self-)standards and the associated fear of failure. Such a space for reflection would thus have to be part of a preparatory measure, as suggested by both our research and other studies [63, 64].

### Limitations

The present study applied a multi-perspective approach by including peer tutors in the survey who had been prepared for either uni- or interprofessional tutorials. In this way, it was possible to gain perspectives from both settings and – in some cases – to juxtapose the two. Although the study and its results are limited to Germany, the findings may nevertheless be interesting for other countries that are currently developing preparatory measures for peer tutors. Our results draw attention to the importance of relationship-building in the context of peer tutoring and tutor preparation, which is also worth noting given different local, regional, and cultural circumstances.

Recruiting participants for this study was challenging, which may have been largely due to the timing of the survey. However, for reasons of survey strategy, no other option was possible. Another reason for this challenge may have been the purposive sampling chosen. Criteria were defined in advance and were held in line with those in the literature (see Appendix A) in order to yield a wide range of different views. Therefore, we decided to include participants from different universities, for which we then conducted online FGD. However, it is possible that this format hindered a more free flowing and engaged discussion from taking place. For example, there was some instances of uncertainty about who wanted to speak and fear of interrupting someone. It is also questionable whether live FGD would really have led to more yield, or whether these effects would not have occurred there due to the lack of familiarity with each other. However, initial indications suggest that online and live discussions do not differ significantly in terms of the content generated [65]. Despite the challenges described beforehand, we achieved the target and literature-recommended number of three discussions [66] as well as the recommended number of participants per group [67]. This enabled us to gain rich data. Data saturation was not aimed for in this study, though it is not possible to rule out whether further FGD would have provided additional insights on the topic.

The experiential orientation of the thematic analysis allowed data to be interpreted with a focus on the peer tutors’ perspectives from both a group and an individual point of view. This flexibility enabled data to be analysed inductively for both semantic and latent meanings. Furthermore, the different perspectives of the peer tutors for uniprofessional and interprofessional tutorials were able to be included in the analysis in roughly equal proportions. In this way, it was possible to gain first insights into a field of action in medical and health professions education that has not yet been well researched. Due to the design, nature, and conditions of the study, our research results are not generalisable and can only be transferred to other contexts to a limited extent. However, these results may point to perspectives for future research activities on the topic. A completed COREQ checklist can be found in supplement B.

## Conclusions

Our results demonstrate that peer tutor preparation practices in uni- and interprofessional settings go beyond merely delivering content and thus also measurable learning outcomes. Instead, these practices play a vital role in shaping the dynamics and relations within peer tutorials at multiple levels, including in shaping social and cognitive congruence among tutors and tutees. Hence, tutor preparation can at least confound the main impact mechanisms of PAL. The striking importance of relationship-building should be emphasised, and all educators who plan to – or who currently – implement tutor preparation or training should be aware of this importance. In order to reach the goal of needs-based preparatory measures, participatory approaches that actively involve different student groups are recommended.

Greater focus on the relational aspects of this learning format than on content and efficacy should lead to increased interaction with less hierarchical thinking and more trust-building. There is still a need for more detailed pedagogical reflection and discussion among medical and health professions educators both on preparatory activities for peer tutors and on the possible impact of these activities. In this respect, further research is warranted in order to identify preparatory measures that promote relation-building and that thus ultimately influence the overall PAL experience positively.

## Electronic supplementary material

Below is the link to the electronic supplementary material.

Supplementary Material 1

Supplementary Material 2

## Data Availability

The datasets used and/or analysed during the current study are available from the corresponding author on reasonable request.

## Appendix A: deductive sampling strategy

**Table:**

Criterion	Decision	Justification
Gender: m/f/d	Heterogeneous	Gender-specific conversation dynamics are known, but they are not likely to have any impact in terms of content. The aim is to not make comparisons that could be influenced by gender [67, p. 81].
Age in years	Heterogeneous: at least of legal age; otherwise, no restriction	Although no significant age differences are expected due to the fact that the discussions take place with people currently in – or recently finished with – their studies, the following applies if there are “outliers”: More life experience can enrich the discussion by shaking up one’s own perspective and thus forcing it to be reconsidered [32].
Students in a health profession (medicine, nursing, PT, OT, SLT, midwifery, or another specialisation)	Heterogeneous: at least two different disciplines Homogeneous: current/former students	In order to take the interprofessional aspect into account in the discussions; according to the definition of interprofessional education, at least two different disciplines are required [68]. A homogeneity status is recommended [66] and is understood here as “students”.
Experience and preparation of peer tutors	Homogeneous in experience / experience as a peer tutor (whether for uni- or interprofessional contexts) AND at least one preparatory meeting of at least 60 min and up to several hours of training	The group should be as homogeneous as possible in terms of language and thematically related knowledge level [66]. Different information on preparation is known: There are structured trainings, but not every preparation is a form of training. Short talks/introductions can also serve as preparation.
Institutions: universities & universities of applied sciences	Universities & universities of applied sciences (not vocational schools in the health sector)	Universities may be easier to reach through their research and are open to research projects. The concept of tutorials in the literature is mostly known from higher education institutions. Herinek et al. (2022a) [30] also revealed that interprofessional tutorials are mainly found in the tertiary education sector.
Artificial vs. real groups: group members are not known to one another	Artificial	Due to their heterogeneous professional composition, real groups are more difficult to organise; moreover, an open exchange of opinions and diverse discussions are more likely in artificial groups [69]. There are no pre-determined hierarchies / role patterns [66, p. 77], and there is no general “common sense” of the group [70, p. 107 ff.; 71, p. 297 f.].

## Appendix B

### Statement on reflexivity

The experience and involvement of the first author, DH, is briefly summarised here. DH was a peer tutor herself 12 years ago. She ran peer tutorials in anatomy/physiology and psychology for a total of 2 years.

A few tutors were known to the first author from university work contexts and IPE networks. However, at no time were there any close professional or personal working relationships with them that would have had an influence on the course of the FGD.

None of the assistants had experience as a peer tutor nor did they know the peer tutors personally.

Throughout the whole process, the co-authors repeatedly encouraged reflection in order to avoid bias when analysing the data.

### Overview of the phase-by-phase analysis in line with Braun/Clarke (2022)[37], including the proportionate task processing^2^

The first phase involved familiarisation with the data. The transcripts were read several times, and the audio data were listened to again in parallel. During this process, notes were written on passages that were relevant to the research question, and initial “loose” ideas and thoughts that related to the three individual group discussions as well as to the entire dataset were written down.

Inductive coding was then applied. This process of marking and briefly describing important passages in the text was carried out twice on the entire dataset: First, one researcher (DH) coded all of the material, and another (VH) coded only one of the transcripts. The codings were next compared and discussed (DH/VH). Only then was the coding repeated and continued. After the initial co-coding process had been completed, the two researchers summarised the codes in dialogue.

The third phase involved the initial identification of themes based on the co-design process. The different code labels were first abstracted by one researcher (DH) by taking into account overlaps in content and strong interdependencies. This process enabled the first themes to be identified. According to Braun/Clarke (2022), themes can be distinguished from one another and involve a central idea, often in the form of a common or important pattern of meaning. Themes may contain sub-themes that describe an aspect of the theme in greater detail.

Thus, after the initial themes had been developed, these “candidate themes” were reviewed in the research tandem (DH/VH) regarding their relevance to the research question. If themes were duplicated or missing, they were deleted or added, respectively. For this purpose, all transcripts were re-read, and the coded passages and text segments were checked for the characteristics of the above-mentioned themes and then revised accordingly.

In the fifth phase, the themes were carefully evaluated by writing a definition for each theme after checking its plausibility and delimitability as well as its interrelationships with other themes. Finally, the themes were named (DH/VH).

The written preparation marks the sixth and final phase of analysis, in which the themes and their respective interpretations were presented.

## Footnotes

1 The quotations that are used in this results section were translated into English by DH and checked by a professional translator and proofreader.

## Declarations

### Proofread

The manuscript underwent professional proofreading.

### Clinical trial number

Not applicable, as this is not a clinical study.

## Abbreviations

FGD: Focus group discussion(s)
PAL: Peer-assisted learning
Prep4TUT: Preparation for a tutoring activity for (interprofessional) Peer–assisted learning-Influences on an interactive teaching and learning format
TA: Thematic Analysis
