# Supplementary material for: “Between formulas and freestyle” – a qualitative analysis of peer tutor preparation and its impact on peer relations
Source: BMC Med Educ. 2024 Oct 19;24:1173. doi: 10.1186/s12909-024-06191-7 (PMC11490185; doi:10.1186/s12909-024-06191-7)
Supplement: Supplementary file 1 — Supplementary Material 1 [file 12909_2024_6191_MOESM1_ESM.pdf]

Interview Guide – English version

Time	Phase	Questions	If necessary, follow-up / in-depth questions / statements	Expectations
**0–5 min**	*Opening questions*	You are all here today because you are tutors (at your universities) in different contexts, for different professions, and with different focuses. Let's begin with a round of introductions. Who are you, what are you studying, and what semester are you in? Also, what subject(s) do you tutor?<br><br>And what is your personal "superpower"?	"Superpower" means a personal strength or ability.	Learn about participants; make different contexts transparent for everyone<br><br>Superpower as a personality trait/ability (can also be important for course or dynamics of GD for me) as icebreaker
**5–10 min**		How would finish the following sentence: "I am a tutor because…"?		Clarify motivation
**10–30 min**	*Introductory questions*	I would find it particularly exciting to learn whether and – if so – how exactly you were prepared for this task. Can you please tell me about it?	○ Maybe one or two of you became active yourselves. Tell me about how you prepared yourselves.<br>○ For those who may have done interprofessional tutorials before, how was the preparation? Did your	As comprehensive a presentation of current preparation practices as possible<br><br>- Type of preparation (external)

Time	Phase	Questions	If necessary, follow-up / in-depth questions / statements	Expectations
			preparation differ from the preparation used for these other tutorials?	- Type of preparation (personal)<br>- Differences in types of preparation for IPE
Now, I would like to ask you about the impact of your preparation.				
**30–45 min**	*Transition questions*	**Now, you (all) have gone through different types of preparation. Can you tell me something about the impact of this preparation?**	**To what extent did your preparation have an impact on or change something about…**<br>○ **… your actions as a tutor / the way you run tutorials?**<br>○ **… how you interact with one another?**<br>○ **… the quality of the joint processing of learning tasks or problem solving?**<br>○ **… how you work with others as tutors now?**<br>○ **… your own understanding of your role?**	**Influence on impact factors**<br><br>**Experiences regarding changes in own actions/behaviour, peers' actions/behaviour, interaction with one another (tutor & peers)**
**45–50 min**		**At this point, those who have already conducted interprofessional tutorials should respond. How do**	**To what extent did your preparation have an impact on…**	**Influence on impact factors**

Time	Phase	Questions	If necessary, follow-up / in-depth questions / statements	Expectations
		**you see the situation, and what experiences have you had with the effects or changes brought about by the preparation?**	○ **… your actions as a tutor / the way you run tutorials?**<br>○ **… how you interact with one another?**<br>○ **… the quality of the joint processing of learning tasks or problem solving?**<br>○ **… how you work with others as tutors now?**<br>○ **… your own understanding of your role?**	**Experiences regarding changes in own actions/behaviour, peers' actions/behaviour, interaction with one another (tutor & peers) in IPE (with more differences between peers and different requirements for tutor).**
Thank you for sharing your personal experiences. I would now like to discuss the necessity of tutor preparation with you in a more general and fundamental way. For that purpose, I will present you with statements that you are kindly asked to state your position on.				
**50–65 min**	*Key questions*	**There are experts who think that specific tutor preparation is indispensable. Others think that preparation is not necessary and even think that preparation may have undesirable effects. What do you think about that?**<br><br>*[undesirable effects = e.g. the influence on tutors is very strong, and changes are thus made to (joint) learning].*	○ **Which thesis (i.e. preparation is absolutely necessary vs. preparation should not take place at all) appeals to you more, and why?**<br>○ **What do you think about interprofessional tutorials?**<br>○ **Do tutors in the IPE context need other/special preparation for their tasks? If so, what kind?**	**Positions and arguments for and against tutor preparation.**<br><br>**Positions and arguments for and against tutor preparation in IPE context.**

Time	Phase	Questions	If necessary, follow-up / in-depth questions / statements	Expectations
			○ **How should such a measure look / be designed so that IP can be promoted?**	
**65–80 min**		**I would now like to ask you a question to think about based on the following quotation:**<br><br>***"If I am qualified to tutor, then I am no longer a peer to those I tutor."*** (Trimbur J 1987: 23)]<br><br>**What do you think about this statement?**	○ **What consequences would the truth value of this quotation have for you?**	**Effects of preparation/influence on impact factors**<br><br>**Opinions on what preparation does / can do.**
We are now slowly coming to the end of the discussion, so it's time to draw some conclusions. I am interested in what you have to say.				
**80–90 min**	*Closing questions*	**As you think back on the discussion so far, how would you personally summarise it?**	○ **What was the most important aspect of the discussion for you?**<br>○ **Does peer-assisted learning require preparation? If so, what aspect would be most important to you?**<br>○ **Regarding interprofessional peer-assisted learning, is there any special**	**Summary**<br><br>**Concluding positions on necessity & impact of preparation and specifics of IPE.**<br><br>**Assessment of what is most important.**

Time	Phase	Questions	If necessary, follow-up / in-depth questions / statements	Expectations
			preparation needed? If so, what do you think about it?	
90–95 min		Is there anything else you would like to add at this point? Maybe something we haven't talked about yet but that is important to you?		Option to make final statement
Thanks & farewell				

Interview Guide – German version

Zeit	Phase	Impuls	Ggf. Nachfragen / Vertiefungsfragen	Erwartete Reaktionen
**0-5 min**	*Opening questions*	**Ihr seid heute alle hier, weil Ihr (an Euren Hochschulen) als Tutor:innen in verschiedenen Kontexten, für verschiedene Berufe und mit verschiedenen Schwerpunkten tätig seid. Lasst uns mit einer Vorstellungsrunde beginnen. Wer seid ihr, was studiert ihr und in welchem Semester seid ihr? Für was seid ihr Tutoren?**<br><br>**und was ist Eure persönliche „Superkraft“?**	**Superkraft meint hier eine persönliche Stärke bzw. Fähigkeit.**	**Fakten über Personen; verschiedene Kontexte für alle transparent machen;**<br><br>**Superkraft als Persönlichkeitsmerkmal / Fähigkeit o. Ä. (was ja auch für den Verlauf/die Dynamik einer GD für mich wichtig sein kann) als Eisbrecher**
**5-10 min**		**Wenn ihr den Satz „Ich bin Tutor, weil…“ beenden müsstet – wie würdet ihr antworten?**		**Motivationsklärung**
**10-30 min**	*Introductory questions*	**Spannend finde ich vor allem, ob - und wenn ja - wie genau Ihr auf diese Aufgabe vorbereitet wurdet. Könnt ihr mir bitte davon berichten?**	○ **Vielleicht wurde der/die eine oder andere ja auch selbst aktiv – erzählt mir mal davon, wie Ihr Euch selbst vorbereitet habt.**	**Möglichst umfassende Darstellung der derzeitigen Vorbereitungspraxis**

<table>
<tr><th>Zeit</th><th>Phase</th><th>Impuls</th><th>Ggf. Nachfragen / Vertiefungsfragen</th><th>Erwartete Reaktionen</th></tr>
<tr><td></td><td></td><td></td><td>○ Diejenigen, die vielleicht schon interprofessionelle Tutorien durchgeführt haben – wie lief die Vorbereitung da ab? Hat sich da die Vorbereitung unterschieden?</td><td>- Art der Vorbereitung (extern)
- Art der Vorbereitung (persönlich)
- Unterschiede der Art der Vorbereitung bei IPE</td></tr>
<tr><td colspan="5">Jetzt möchte ich Euch zu den Auswirkungen dieser Vorbereitungen befragen.</td></tr>
<tr><td>30-45 min</td><td>Transition questions</td><td>Nun habt ihr (alle) unterschiedliche Vorbereitungen durchlaufen. Könnt Ihr mir etwas über die Auswirkungen der Vorbereitungen sagen?</td><td>Inwiefern hatte die Vorbereitung Auswirkungen…etwas verändert…
○ auf euer Handeln als Tutor?/Die Art wie Ihr Tutorien durchführt?
○ darauf, wie ihr miteinander interagiert…
○ auf die Qualität der der gemeinsamen Bearbeitung von Lernaufgaben bzw. Lösung von Problemen?
○ Wie arbeitet ihr jetzt als Tutoren mit anderen zusammen?
○ Auf euer eigenes Rollenverständnis?</td><td>Einfluss auf Wirkfaktoren

Erfahrungen in Bezug auf Veränderungen im eigenen Handeln/Verhalten, dem Handeln/Verhalten der Peers, der Interaktion untereinander (Tut & Peers)</td></tr>
</table>

Zeit	Phase	Impuls	Ggf. Nachfragen / Vertiefungsfragen	Erwartete Reaktionen
**45-50 min**		**Wenn sich an dieser Stelle jetzt noch mal diejenigen, die bereits interprofessionelle Tutorien durchgeführt haben, zu Wort melden. Wie stellt sich die Situation bei Euch dar? / Welche Erfahrungen habt Ihr gemacht mit den Auswirkungen bzw. Veränderungen durch die Vorbereitung gemacht?**	**Inwiefern hatte die Vorbereitung Auswirkungen…**<br>○ **auf euer Handeln als Tutor?/Die Art wie Ihr Tutorien durchführt?**<br>○ **darauf, wie ihr miteinander interagiert…**<br>○ **auf die Qualität der der gemeinsamen Bearbeitung von Lernaufgaben bzw. Lösung von Problemen?**<br>○ **Wie arbeitet ihr jetzt als Tutoren mit anderen zusammen?**<br>○ **Auf euer eigenes Rollenverständnis?**	**Einfluss auf Wirkfaktoren**<br><br>**Erfahrungen in Bezug auf Veränderungen im eigenen Handeln/Verhalten, dem Handeln/Verhalten der Peers, der Interaktion untereinander (Tut & Peers unter der Besonderheit IPE (mit mehr Differenzen zw. den Peers und anderen Anforderungen für die Tut.)**
Danke für Eure persönlichen Erfahrungen. Ich würde nun gern mit euch etwas allgemeiner bzw. grundsätzlicher über die Notwendigkeit der Vorbereitung von Tutor:innen diskutieren. Dafür werde ich Euch Aussagen präsentieren, zu denen ihr bitte Eure Position vertretet.				
**50-65 min**	*Key Questions*	**Es gibt Experten, die halten eine gezielte Vorbereitung für Tutor:innen für unverzichtbar. Andere meinen, dass eine Vorbereitung nicht nötig sei – sie denken sogar, dass die Vorbereitung vielleicht sogar unerwünschte Effekte hat. Was denkt Ihr darüber?**	○ **Welche These (Vorbereitung zwingend nötig vs. Vorbereitung sollte gar nicht stattfinden) sagt euch mehr zu und warum?**<br>○ **Was denkt ihr hier, wenn es um interprofessionelle Tutorien geht?**	**Positionen und Argumente für bzw. gegen die Vorbereitung von Tutor:innen.**<br><br>**Positionen und Argumente für bzw. gegen die Vorbereitung von**

Zeit	Phase	Impuls	Ggf. Nachfragen / Vertiefungsfragen	Erwartete Reaktionen
		[unerwünschte Effekte = z. B. Einfluss auf die Tutoren sehr groß und dadurch Veränderungen im (gemeinsamen) Lernen]	○ **Brauchen Tutor:innen im IPE Kontext andere/besondere Vorbereitung auf Ihre Aufgaben? Und wenn ja, welche?**<br>○ **Wie sollte eine solche Maßnahme aussehen/gestaltet sein, damit IP gefördert werden kann?**	**Tutor:innen im IPE Kontext.**
**65-80 min**		**Ich möchte euch nun eine Frage zum Nachdenken stellen. Dafür habe ich ein Zitat mitgebracht:**<br><br>***„Wenn ich für meine Aufgabe als Tutor qualifiziert wurde, kann ich eigentlich kein (gleichberechtigter) Peer für die anderen Studierenden mehr sein.“***<br><br>“***If I am qualified to tutor, then I am no longer a peer to those I tutor. […]”*** (Trimbur J 1987: 23)]<br><br>**Wie denkt Ihr über diese Aussage?**	○ **Was hätte das für Konsequenzen für Euch?**	**Wirkungen der Vorbereitungen / Einfluss auf Wirkfaktoren**<br><br>**Meinungen zu dem, was Vorbereitung bewirkt / bewirken kann.**
Wir kommen nun langsam zum Ende der Diskussion. Daher wird es Zeit ein Fazit zu ziehen. Ich bin interessiert an eurem Fazit.				

Zeit	Phase	Impuls	Ggf. Nachfragen / Vertiefungsfragen	Erwartete Reaktionen
**80-90 min**	*Ending Questions*	**Wenn Ihr Euch die bisherige Diskussion noch einmal in Erinnerung ruft - was ist Euer persönliches Resümee?**	○ **Was war für Euch der wichtigste Aspekt?**<br>○ **Braucht es für das Peer-assisted Learning eine Vorbereitung? Wenn ja, welcher Aspekt wäre Euch dabei am wichtigsten?**<br>○ **Wie verhält es sich beim interprofessionellen Peer-Assisted-Learning – wird dafür eine spezielle Vorbereitung benötigt? Was denkt Ihr darüber?**	**Resümee**<br><br>**Abschließende Positionen zur Notwendigkeit, den Auswirkungen der Vorbereitungen und Besonderheiten von IPE.**<br><br>**Einschätzung dessen, was am wichtigsten ist.**
**90-95 min**		**Gibt es noch etwas, das ihr an dieser Stelle gern ergänzen möchtet – etwas über das wir noch nicht gesprochen haben, das Euch aber wichtig ist?**		**Möglichkeit, sich abschließend zu äußern**
**DANK & Abschied**				
